# Sex disparities in systemic sclerosis-associated pulmonary arterial hypertension: a cohort study

**DOI:** 10.1186/s13075-016-0933-1

**Published:** 2016-01-27

**Authors:** Christopher R. Pasarikovski, John T. Granton, Adrienne M. Roos, Saghar Sadeghi, Amie T. Kron, John Thenganatt, Jakov Moric, Cathy Chau, Sindhu R. Johnson

**Affiliations:** Toronto Scleroderma Program, Mount Sinai Hospital, Toronto Western Hospital, Division of Rheumatology, Department of Medicine, Faculty of Medicine, University of Toronto, Ground Floor, East Wing, Toronto Western Hospital, 399 Bathurst Street, Toronto, ON M5T 2S8 Canada; University Health Network Pulmonary Hypertension Programme, Toronto General Hospital, Divisions of Respirology and Critical Care Medicine, Department of Medicine, Faculty of Medicine, University of Toronto, Toronto, ON Canada; University Health Network Pulmonary Hypertension Programme, Toronto General Hospital, Division of Respirology, Department of Medicine, Faculty of Medicine, University of Toronto, Toronto, ON Canada; University Health Network Pulmonary Hypertension Programme, Toronto General Hospital, Division of Respirology, Women’s College Hospital, Department of Medicine, Faculty of Medicine, University of Toronto, Toronto, ON Canada; Institute of Health Policy, Management and Evaluation, University of Toronto, Toronto, ON Canada

**Keywords:** Systemic sclerosis, Scleroderma, Pulmonary arterial hypertension, Sex, Survival, Scleroderma renal crisis

## Abstract

**Background:**

The impact of male sex as a determinant of health outcomes in systemic sclerosis-associated pulmonary arterial hypertension (SSc-PAH) is controversial. The primary objective of this study was to evaluate the effect of sex on survival in patients with SSc-PAH. The secondary objectives were to evaluate the effect of sex on age of PAH diagnosis, time from SSc diagnosis to PAH diagnosis, and SSc disease manifestations.

**Methods:**

Sex-based disparities were evaluated in a cohort of SSc-PAH patients with a primary outcome of time from PAH diagnosis to all-cause mortality. Secondary outcomes were differences in age of diagnosis, disease duration, and SSc manifestations. Survival differences were evaluated using Kaplan-Meier and Cox proportional hazard models.

**Results:**

We identified 378 SSc-PAH (58 males, 320 females) patients, with a female:male ratio of 5.5:1. Males had a shorter mean ± standard deviation time from SSc diagnosis to PAH diagnosis (1.7 ± 14 versus 5.5 ± 14.2 years); shorter PAH duration (3.5 ± 3.1 versus 4.7 ± 4.2 years), increased frequency of renal crisis (19 % versus 8 %, relative risk (RR) 2.33, 95 %CI 1.22, 4.46), interstitial lung disease (67 % versus 48 %, RR 1.41, 95 %CI 1.14, 1.74), and diffuse subtype (40 % versus 22 %, RR 1.84, 95 %CI 1.26, 2.69). Males appeared to have decreased 1-, 2-, 3-, and 5-year survival (83.2 %, 68.7 %, 53.2 %, 45.6 %) compared to females (85.7 %, 75.7 %, 66.4 %, 57.4 %). However, there was no difference in mortality between sexes (HR 1.43 (95 %CI 0.97, 2.13).

**Conclusions:**

Sex disparities appear to exist in the frequency of PAH, time to PAH diagnosis, PAH disease duration and SSc disease burden. However, male sex does not independently impact SSc-PAH survival.

## Background

Systemic sclerosis (SSc) is a systemic autoimmune rheumatic disease characterized by vasculopathy and fibrosis that primarily affects females with a female:male ratio 3–4:1 [1]. Sex hormones likely play an important role in the observed female preponderance in SSc [2]. Although female prevalence is high, several authors have reported that male sex is associated with decreased survival [3–7]. Male sex has been associated with an increased risk of mortality in SSc patients above that observed for males in the general population [7].

SSc-associated pulmonary arterial hypertension (SSc-PAH) is a leading cause of SSc-related mortality, with a prevalence of approximately 7 % [8]. Little is known about the effect of sex on disease onset, time to PAH diagnosis, and survival in patients with SSc-PAH [9, 10]. In a cohort study of 259 SSc-PAH patients, male sex was associated with increased risk of mortality (hazard ratio (HR) 2.2, 95 % confidence interval (CI) 1.35, 3.55) [11]. However, in another cohort study of 152 SSc-PAH patients, male sex was not an independent risk factor for mortality (HR 2.02, 95 %CI 0.65, 6.2) [12]. Furthermore, sex was not an independent risk factor in a cohort of patients with connective tissue disease-associated PAH (22 % of whom had SSc-PAH) and idiopathic PAH (IPAH) (HR 0.78, 95 %CI 0.40, 1.51) [13]. Using data from the REVEAL registry, Benza et al. found that men >60 years of age had poorer 1-year survival compared to females (HR 2.2, 95 %CI 1.6, 3.0) [14]. This finding was confirmed by Shapiro et al., who reported sex differences in 2-year survival from enrolment in the REVEAL registry among men diagnosed with group I PAH aged >60 years (HR 1.67, 95 %CI 1.28, 2.17) [15]. They also noted that male sex was associated with poorer survival for the IPAH subgroup of group I PAH patients across all age groups [15]. Humbert et al. reported that among patients with IPAH, familial, and anorexigen-associated PAH, females had better survival (HR 0.52, 95 %CI 0.30, 0.88 [16]. However, Fisher et al. reported that there was no significant difference between males and females in 3-year survival for patients diagnosed with IPAH and SSc-PAH combined [17].

Thus, the current literature on the effect of sex on SSc-PAH is limited and conflicted. The effect of sex on age of onset, time to diagnosis, disease duration, disease manifestations and survival in SSc-PAH is not well understood. The aim of this study is to improve our understanding of the sex-based disparities in SSc-PAH. The primary objective of this study was to evaluate the effect of sex on survival in patients with SSc-PAH. The secondary objectives were to evaluate the effect of sex on age of PAH diagnosis, time from SSc diagnosis to PAH diagnosis, and SSc disease manifestations.

## Methods

### Patients

The University Health Network Pulmonary Hypertension Programme has the largest published single-center longitudinal cohort in Canada [18]. Patients are prospectively followed every 6 to 12 months using a standardized protocol. Adult SSc-PAH patients seen between 1998 and January 1, 2014 were included if they fulfilled the American College of Rheumatology (ACR) – European League Against Rheumatism (EULAR) classification criteria for SSc [19], and had PAH confirmed by catheterization with a mean pulmonary artery pressure (mPAP) >25 mm Hg, pulmonary capillary wedge pressure (PCWP) <15 mm Hg and pulmonary vascular resistance (PVR) >3 Wood units on catheterization [20]. Patients with another etiology for PAH (e.g. human immunodeficiency virus, anorexigen use, portal hypertension, congenital cardiac abnormalities) were excluded.

### Exposure

Sex was defined as self-reported biological and physiological characteristics at birth, and characterized as male or female. Gender (roles, behaviours, activities, and attributes that a given society considers appropriate) was not assessed. Patients were excluded from analysis if they had a known history of sex chromosome abnormalities or had undergone sex reassignment surgery.

### Outcome

The primary outcome of the study was the time from PAH diagnosis to death from all causes. Patients who were alive as of June 1, 2014 were censored. PAH diagnosis was defined as the date of right heart catheterization. SSc diagnosis was defined as the date a diagnosis of SSc was made by a physician. Dates of death were obtained from the clinic chart, hospital electronic record or obituary. Secondary outcomes included sex differences in age at PAH diagnosis, time from SSc diagnosis to PAH diagnosis, PAH disease duration, subtype of SSc (limited, diffuse), and SSc manifestations (Raynaud’s phenomenon, digital ulceration, esophageal dysmotility, telangiectasia, interstitial lung disease (bibasilar reticular abnormalities with minimal ground-glass on high-resolution computerized tomography (CT) of the thorax) and serology (centromere, topoisomerase I (ScL-70); RNA polymerase III was not available). Comorbidities (coronary artery disease, hypertension, diabetes, hyperlipidemia, atrial fibrillation, and stroke), medications (warfarin, calcium channel blocker, endothelin receptor antagonist, prostaglandin analogue, and phosphodiesterase inhibitors), pulmonary function and hemodynamic measurements were compared.

### Analysis

All statistical analysis was done using RStudio (version 0.98.932, R Foundation for Statistical Computing, 2012). The Shapiro-Wilk statistic was used to test for normality. Continuous data were not normally distributed. Baseline characteristics for males and females were analyzed using standardized differences, and a difference of 25 % was considered significant. Use of standardized differences is a method of comparing two groups, independent of sample size. For dichotomous variables we also reported the relative risk (RR). Survival data were plotted using Kaplan-Meier curves and significance was tested using the log-rank test. Independent predictors of survival were evaluated using Cox regression analysis. Hazard ratios (HR) are reported with 95 % confidence intervals (95 % CI). University Health Network (12-5253-AE) and Mount Sinai Hospital (12-0233-C) research ethics board approvals were obtained for the conduct of this study. The ethics boards waived the need for consent, as this was a retrospective study with a high mortality rate.

## Results

### SSc-PAH patient characteristics

A total of 1142 charts were reviewed and 378 SSc-PAH patients were identified of whom 58 (15.3 %) were male and 320 (84.7 %) were female. All patients fulfilled the ACR-EULAR classification criteria for SSc. The female:male ratio was 5.5:1. The mean age at diagnosis was 54.5 ± 11.0 years for males and 57.3 ± 13.0 years for females. Males had a shorter mean time from SSc diagnosis to PAH diagnosis (1.7 ± 14 versus 5.5 ± 14.2 years; standardized difference 93 %) and shorter PAH disease duration (3.5 ± 3.1 versus 4.7 ± 4.2 years, standardized difference 29 %). Males had an increased frequency of renal crisis (19 % versus 8 %, relative risk (RR) 2.33, 95 %CI 1.22, 4.46), interstitial lung disease (67 % versus 48 %, RR 1.41, 95 %CI 1.14, 1.74) and diffuse cutaneous disease (40 % versus 22 %, RR 1.84, 95 %CI 1.26, 2.69). Adjusting for the presence of interstitial lung disease had marginal to no effect on the effect of male sex on pulmonary hypertension age of diagnosis, time from SSc diagnosis to PAH diagnosis and pulmonary hypertension disease duration. There were no significant differences between males and females in the presence of Raynaud’s phenomenon, telangiectasia, digital ulcers, esophageal dysmotility, serology, 6-minute walk distance, World Health Organization (WHO) functional class, brain natriuretic peptide levels, comorbidities and medications (Table 1).

**Table 1:** SSc-PAH baseline characteristics by sex

SSc patient characteristics	Males	Females	Standardized difference
	n = 58	n = 320	
Age at PAH diagnosis, y, mean (SD)	54.5 (11.0)	57.3 (13.0)	0.05
Time from SSc to PAH diagnosis, y, mean (SD)	1.7 (14.0)	5.5 (14.2)	0.93^a^
PAH disease duration, y, mean (SD)	3.5 (3.1)	4.7 (4.2)	0.29^a^
SSc manifestations			
Diffuse subtype, n (%)	23 (40 %)	69 (22 %)	0.40^a^
Raynaud’s phenomenon, n (%)	56 (97 %)	302 (94 %)	0.10
Telangiectasia, n (%)	46 (79 %)	224 (70 %)	0.22
Renal crisis, n (%)	11 (19 %)	26 (8 %)	0.32^a^
Esophageal dysmotility, n (%)	51 (88 %)	276 (86 %)	0.05
Digital ulcers, n (%)	16 (28 %)	120 (38 %)	0.21
Interstitial lung disease, n (%)	39 (67 %)	153 (48 %)	0.40^a^
ScL-70 antibody, n (%)	10 (17 %)	36 (11 %)	0.17
Anticentromere antibody, n (%)	9 (15 %)	49 (17 %)	0.06
Cardiopulmonary measures			
6MWD, m, mean (SD)	344.3 (140.9)	324.5 (154.6)	0.06
WHO functional class III-IV, n (%)	24 (41 %)	124 (39 %)	0.05
mPAP, mmHg, mean (SD)	44.9 (20.5)	40.7 (21.7)	0.10
mPVR, dyn · s/cm^5^, mean (SD)	705.0 (710.5)	584.7 (552.9)	0.19
Cardiac output L/min, mean (SD)	4.2 (2.4)	3.3 (1.6)	0.26^a^
PCWP, mmHg, /mean (SD)	13.3 (6.2)	9.5 (6.2)	0.33^a^
BNP pg/mL, mean (SD)	203.0 (266.9)	245.9 (481.3)	0.19
FVC, % predicted	79.3 (17.4)	76.1 (24.6)	0.04
FEV1, % predicted	80.3 (18.2)	78.2 (26.1)	0.03
DLCO, ml/min/mmHg	52.1 (19.7)	55.3 (19.5)	0.06
Right ventricular enlargement			
Normal	40 (69 %)	250 (78 %)	0.21
Mild	5 (9 %)	24 (8 %)	0.04
Moderate	10 (17 %)	35 (11 %)	0.18
Severe	3 (5 %)	11 (3 %)	0.09
Right ventricular dysfunction			
Normal	39 (67 %)	249 (78 %)	0.23
Mild	5 (9 %)	26 (8 %)	0.02
Moderate	12 (21 %)	38 (12 %)	0.24
Severe	2 (3 %)	7 (2 %)	0.07
Comorbidities			
Coronary artery disease, n (%)	12 (21 %)	40 (13 %)	0.22
Hypertension, n (%)	13 (22 %)	88 (28 %)	0.12
Diabetes mellitus, n (%)	5 (9 %)	21 (7 %)	0.08
Hyperlipidemia, n (%)	8 (14 %)	26 (8 %)	0.18
Atrial fibrillation, n (%)	6 (10 %)	28 (9 %)	0.05
Peripheral vascular disease, n (%)	3 (5 %)	19 (6 %)	0.03
Stroke, n (%)	2 (3 %)	15 (5 %)	0.06

*SSc-PAH* systemic sclerosis-associated pulmonary arterial hypertension, *ScL-70* topoisomerase I, *6MWD* 6-minute walk test, *WHO* World Health Organization, *mPAP* mean pulmonary artery pressure, *mPVR* mean pulmonary vascular resistance, *PCWP* pulmonary capillary wedge pressure, *BNP* brain natriuretic peptide, *FVC* forced vital capacity, *FEV1* forced expiratory volume 1, *DLCO* diffusing capacity of the lungs for carbon monoxide

^a^Denotes standardized difference greater than 25 %

### SSc-PAH survival

**Fig. 1:**
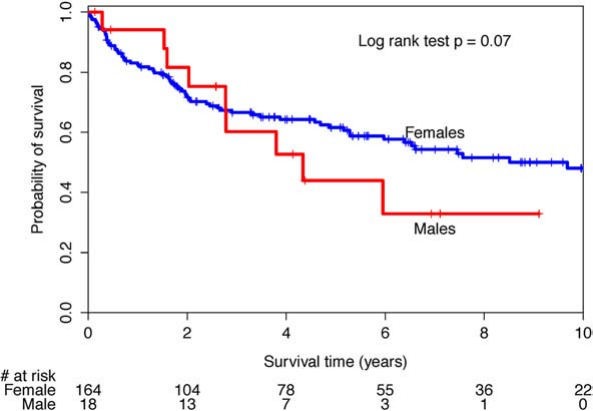
Kaplan-Meier survival curves for systemic sclerosis-associated pulmonary arterial hypertension (SSc-PAH) cohort by sex

There were 32 deaths (55 %) among males and 155 deaths (48 %) among females. The 1-, 2-, 3-, and 5-year survival estimates were 83.2 %, 68.7 %, 53.2 %, 45.6 % for males, and 85.7 %, 75.7 %, 66.4 %, 57.4 % for females (Table 2). The unadjusted median survival time for males was 3.8 years compared to a median survival of 6.5 years for females. There was no significant difference in Kaplan-Meier survival curves, log-rank test *p* = 0.07 (Fig. 1). The unadjusted HR for the effect of male sex on survival was 1.43 (95 %CI 0.97, 2.13). After adjusting for baseline differences disease subtype and interstitial lung disease, the HR for male sex was attenuated to 1.27 (95 %CI 0.85, 1.90).

**Table 2:** SSc-PAH survival estimates by sex

Sex	Survival				
	1-year	2-year	3-year	5-year	Median
	(95 %CI)	(95 %CI)	(95 %CI)	(95 %CI)	years
Male	83.2 %	68.7 %	53.2 %	45.6 %	3.8
	(73.8 %, 93.9 %)	(57.1 %, 82.8 %)	(40.7 %, 69.4 %)	(33.1 %, 62.8 %)	
Female	85.7 %	75.7 %	66.4 %	57.4 %	6.5
	(81.8 %, 89.7 %)	(71.0 %, 80.8 %)	(61.7 %, 72.7 %)	(51.7 %, 63.7 %)	

*SSc-PAH* systemic sclerosis-associated pulmonary arterial hypertension

**Fig. 2:**
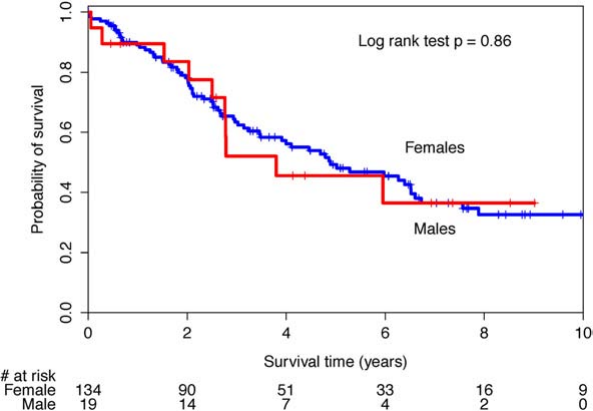
Kaplan-Meier survival curves for systemic sclerosis-associated pulmonary arterial hypertension (SSc-PAH) patients with age greater than 60 at PAH diagnosis by sex

**Fig. 3:**
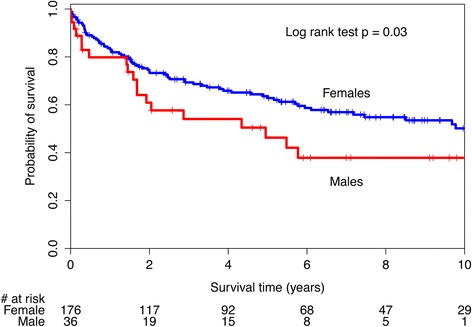
Kaplan-Meier survival curves for systemic sclerosis-associated pulmonary arterial hypertension (SSc-PAH) patients with age less than 60 at PAH diagnosis by sex

A sensitivity analysis was performed to explore the effect of PAH age of diagnosis >60 years that had been reported by others. There was no significant difference in survival curves between this subset of males and females, log-rank test 0.86 (Fig. 2). The unadjusted HR for the effect of male sex on survival in those aged 60 years or older was 1.06 (95 %CI 0.55, 2.07). There was a statistically significant difference in survival curves for those with an age of PAH diagnosis less than 60 years of age (log-rank test *p* = 0.03, Fig. 3) with a male sex unadjusted HR of 1.70 (1.04, 2.80). After adjusting for baseline differences in disease subtype in this subset, the HR for male sex was attenuated to 1.44 (95 %CI 0.85, 2.46).

## Discussion

We have found that sex disparities appear to exist in SSc-PAH. PAH is more common in females with SSc, yet males have a shorter time from SSc to PAH diagnosis, shorter PAH disease duration and increased burden of SSc disease. Despite these differences, male sex does not independently impact SSc-PAH mortality. To our knowledge, this is the largest study to examine the impact of sex in SSc-PAH.

The female to male ratio for SSc-PAH of 5.5:1, is higher than the reported sex ratios for SSc in general, ranging between 3–4:1 [7]. This suggests that PAH is an SSc disease manifestation that occurs more frequently in females. However, males with SSc-PAH have an increased frequency of other serious SSc disease manifestations, notably, scleroderma renal crisis, diffuse cutaneous disease and interstitial lung disease. Furthermore, we found males had a shorter time from SSc diagnosis to PAH diagnosis, with the average male time to SSc-PAH diagnosis more than 3 years earlier than females. In a Japanese cohort study by Kasukawa et al. it was reported that a shortened average time between SSc and SSc-PAH diagnosis was associated with an increase in mortality [21]. Together with our findings, this may suggest that although PAH is less frequent in males, they may have more aggressive disease. The differential time to PAH diagnosis may also be reflective of sex-based differences is health-seeking behaviours and/or access to care [7].

We found that females tended to have better short- and long-term survival rates compared to males. However, sex was not found to be an independent factor for survival in SSc-PAH patients. Condliffe et al. reported that male sex was associated with decreased survival in a cohort of 259 patients with SSc-PAH [11]. MacGregor et al. reported that although male sex was not an independent risk factor, there was a trend toward increased death in male patients with SSc-PAH [12]. In both studies, survival curves for males and females were not provided so graphical comparisons to our findings could not be made. Visual inspection of our Kaplan-Meier curves suggests differential survival between sexes, particularly in the long term. The lack of statistical significance may be related to insufficient power. However, accounting for baseline difference between sexes attenuated the HR toward a null effect. This suggests that perceived differences in survival between sexes in the full cohort are likely attributable to the baseline differences in measured and unmeasured confounders.

When examining SSc-PAH patients with an age of PAH diagnosis greater than 60 years, we found no significant difference in survival between the sexes. Examination of the Kaplan-Meier curves in this subset of patients, illustrates considerable overlap of the survival curves. It has been hypothesized that because post-menopausal female patients have lost the cardio-protective effect of estrogen, right ventricular adaptation is no longer possible. When we evaluated survival in those with an age of PAH diagnosis less than age 60 years, there appeared to be sex-based differences in survival. However the adjusted analysis suggested that these perceived differences were attributable to difference in other baseline characteristics. It may be that the observed differences in SSc-PAH survival between sexes reported in other studies may be attributable to unmeasured confounders.

A potential limitation of this study is the limited power to detect small differences in survival. A larger sample size would allow us to detect if male sex has a small, independent effect on survival. To our knowledge, this is the largest study to evaluate the effect of sex on survival in SSc-PAH, and we had sufficient power to detect moderate to large effects. A second potential limitation is our ability to evaluate all aspects of SSc disease burden. Although we were able to evaluate centromere and ScL-70 antibodies, we did not have the ability to evaluate other scleroderma-specific antibodies including RNA polymerase III. In addition, interstitial lung disease was present in a subset of patients. It should be noted that these patients had mild disease, evidenced by bibasilar reticulations on CT thorax. This may reflect a referral bias at our center as SSc patients with moderate to severe interstitial lung disease, or pulmonary hypertension attributable to interstitial lung disease are not seen on our Pulmonary Hypertension Clinic, but rather in the Interstitial Lung Disease Clinic. As such, patients with moderate to severe interstitial lung disease or pulmonary hypertension attributable to interstitial lung disease were not included in this analysis. Findings from this study should not be generalized to those patients. In addition, we did not collect smoking data. A third potential limitation of our study was the inability to account for sex-based differences in the psycho-social determinants of health outcomes. Our cohort study occurs within the context of a universal health care system, however it is possible that inequitable outcomes may occur. These may be related to sex-based differences in health-seeking behaviors and access to care, which were beyond the scope of this study. Future investigators may consider accounting for these factors. Finally, our survival analysis was limited to all-cause mortality. Since we did not have access to death certificates, we were not able to report cause-specific mortality. However there is controversy regarding the validity of cause-specific mortality obtained from death certificates [22]. As such, our use of all-cause mortality reflects a more conservative approach.

## Conclusions

Sex-based disparities appear to exist in the frequency of PAH, time to PAH diagnosis, PAH disease duration, and SSc disease burden. However, male sex does not independently impact SSc-PAH survival. Similarly, male sex does not independently affect survival in those diagnosed with SSc-PAH after the age of 60. Further research is needed to understand the basis for the differential frequency of PAH and time to diagnosis.

## Abbreviations

95%CI: 95 % Confidence interval
ACR: American College of Rheumatology
CT: Computerized tomography
EULAR: European League Against Rheumatism
HR: Hazard ratio
IPAH: Idiopathic pulmonary arterial hypertension
mPAP: Mean pulmonary artery pressure
PAH: Pulmonary arterial hypertension
PCWP: Pulmonary capillary wedge pressure
PVR: Pulmonary vascular resistance
RR: Relative risk
ScL-70: Topoisomerase I
SSc: Systemic sclerosis
WHO: World Health Organization

## References

[1] Chifflot H, Fautrel B, Sordet C, Chatelus E, Sibilia J (2008). Incidence and prevalence of systemic sclerosis: a systematic literature review. Semin Arthritis Rheum..

[2] Pennell LM, Galligan CL, Fish EN (2012). Sex affects immunity. J Autoimmun..

[3] Mayes MD, Lacey JV, Beebe-Dimmer J, Gillespie BW, Cooper B, Laing TJ (2003). Prevalence, incidence, survival, and disease characteristics of systemic sclerosis in a large US population. Arthritis Rheum..

[4] Hachulla E, Carpentier P, Gressin V, Diot E, Allanore Y, Sibilia J (2009). Risk factors for death and the 3-year survival of patients with systemic sclerosis: the French ItinerAIR-Sclerodermie study. Rheumatology..

[5] Hissaria P, Lester S, Hakendorf P, Woodman R, Patterson K, Hill C (2011). Survival in scleroderma: results from the population-based South Australian Register. Intern Med J..

[6] Kuo CF, Luo SF, Yu KH, Chou IJ, Tseng WY, Chang HC (2012). Cancer risk among patients with systemic sclerosis: a nationwide population study in Taiwan. Scand J Rheumatol..

[7] Hussein H, Lee P, Chau C, Johnson SR (2014). The effect of male sex on survival in systemic sclerosis. J Rheumatol..

[8] Mukerjee D, St George D, Coleiro B, Knight C, Denton CP, Davar J (2003). Prevalence and outcome in systemic sclerosis associated pulmonary arterial hypertension: application of a registry approach. Ann Rheum Dis..

[9] Johnson SR, Swiston JR, Granton JT (2008). Prognostic factors for survival in scleroderma associated pulmonary arterial hypertension. J Rheumatol..

[10] Johnson SR, Granton JT (2011). Pulmonary hypertension in systemic sclerosis and systemic lupus erythematosus. Eur Respir Rev..

[11] Condliffe R, Kiely DG, Peacock AJ, Corris PA, Gibbs JS, Vrapi F (2009). Connective tissue disease-associated pulmonary arterial hypertension in the modern treatment era. Am J Respir Crit Care Med..

[12] MacGregor AJ, Canavan R, Knight C, Denton CP, Davar J, Coghlan J (2001). Pulmonary hypertension in systemic sclerosis: risk factors for progression and consequences for survival. Rheumatology..

[13] Zhang R, Dai LZ, Xie WP, Yu ZX, Wu BX, Pan L (2011). Survival of Chinese patients with pulmonary arterial hypertension in the modern treatment era. Chest..

[14] Benza RL, Miller DP, Gomberg-Maitland M, Frantz RP, Foreman AJ, Coffey CS (2010). Predicting survival in pulmonary arterial hypertension: insights from the Registry to Evaluate Early and Long-Term Pulmonary Arterial Hypertension Disease Management (REVEAL). Circulation..

[15] Shapiro S, Traiger GL, Turner M, McGoon MD, Wason P, Barst RJ (2012). Sex differences in the diagnosis, treatment, and outcome of patients with pulmonary arterial hypertension enrolled in the registry to evaluate early and long-term pulmonary arterial hypertension disease management. Chest..

[16] Humbert M, Sitbon O, Chaouat A, Bertocchi M, Habib G, Gressin V (2010). Survival in patients with idiopathic, familial, and anorexigen-associated pulmonary arterial hypertension in the modern management era. Circulation..

[17] Fisher MR, Mathai SC, Champion HC, Girgis RE, Housten-Harris T, Hummers L (2006). Clinical differences between idiopathic and scleroderma-related pulmonary hypertension. Arthritis Rheum..

[18] Johnson SR, Granton JT, Tomlinson GA, Grosbein HA, Le T, Lee P (2012). Warfarin in systemic sclerosis-associated and idiopathic pulmonary arterial hypertension. A Bayesian approach to evaluating treatment for uncommon disease. J Rheumatol.

[19] van den Hoogen F, Khanna D, Fransen J, Johnson SR, Baron M, Tyndall A (2013). 2013 classification criteria for systemic sclerosis: an American College of Rheumatology/European League against Rheumatism collaborative initiative. Ann Rheum Dis..

[20] Hoeper MM, Bogaard HJ, Condliffe R, Frantz R, Khanna D, Kurzyna M (2013). Definitions and diagnosis of pulmonary hypertension. J Am Coll Cardiol.

[21] Kasukawa R, Nishimaki T, Takagi T, Miyawaki S, Yokohari R, Tsunematsu T (1990). Pulmonary hypertension in connective tissue disease. Clinical analysis of sixty patients in multi-institutional study. Clin Rheumatol.

[22] Johansson LA, Westerling R (2002). Comparing hospital discharge records with death certificates: can the differences be explained?. J Epidemiol Community Health..

